# Application of Immunohistochemistry in the Pathological Diagnosis of Liver Tumors

**DOI:** 10.3390/ijms22115780

**Published:** 2021-05-28

**Authors:** Yoshihisa Takahashi, Erdenetsogt Dungubat, Hiroyuki Kusano, Dariimaa Ganbat, Yasuhiko Tomita, Sarandelger Odgerel, Toshio Fukusato

**Affiliations:** 1 Department of Pathology, School of Medicine, International University of Health and Welfare, Narita, Chiba 286-8686, Japan or erdenetsogt.d@mnums.edu.mn (E.D.); h-kusano@iuhw.ac.jp (H.K.); yasuhiko-tomita@umin.ac.jp (Y.T.); 2 Department of Pathology, School of Biomedicine, Mongolian National University of Medical Sciences, Ulaanbaatar 14210, Mongolia; 3 Department of Public Health, School of Medicine, International University of Health and Welfare, Narita, Chiba 286-8686, Japan; dariimaa.ganbat21@gmail.com; 4 National Pathology Center of Mongolia, Ulaanbaatar 14210, Mongolia; sarandelger0109@gmail.com; 5 General Medical Education and Research Center, Teikyo University, Itabashi-ku, Tokyo 173-8605, Japan; fukusato@med.teikyo-u.ac.jp

**Keywords:** immunohistochemical staining, liver tumor, hepatocellular carcinoma, focal nodular hyperplasia, hepatocellular adenoma, intrahepatic cholangiocarcinoma

## Abstract

Although radiological diagnostics have been progressing, pathological diagnosis remains the most reliable method for diagnosing liver tumors. In some cases, definite pathological diagnosis cannot be obtained by histological evaluation alone, especially when the sample is a small biopsy; in such cases, immunohistochemical staining is very useful. Immunohistochemistry is the most frequently used technique for molecular pathological diagnosis due to its broad application, ease of performance and evaluation, and reasonable cost. The results occasionally reflect specific genetic mutations. The immunohistochemical markers of hepatocellular carcinoma include those of hepatocellular differentiation—such as hepatocyte paraffin 1 and arginase-1—and those of malignant hepatocytes—such as glypican-3, heat shock protein 70, and glutamine synthetase (GS). To classify the subtypes of hepatocellular adenoma, examination of several immunohistochemical markers, such as liver fatty acid-binding protein, GS, and serum amyloid A, is indispensable. Immunohistochemical staining for GS is also important for the diagnosis of focal nodular hyperplasia. The representative immunohistochemical markers of intrahepatic cholangiocarcinoma include cytokeratin (CK) 7 and CK19. In this article, we provide an overview of the application of immunohistochemistry in the pathological diagnosis of liver tumors referring to the association with genetic alterations. Furthermore, we aimed to explain the practical points in the differential diagnosis of liver tumors by immunohistochemical staining.

## 1. Introduction

Although radiological diagnostics have been progressing, pathological diagnosis remains the most reliable method for diagnosing liver tumors, with accurate pathological diagnosis being essential for appropriate treatment. Liquid biopsy has been increasingly used, but traditional biopsy with immunohistochemistry still has its role. Histologic evaluation by microscopic observation of specimens stained with hematoxylin and eosin and other special stains, such as silver stains, is important in the pathological diagnosis of liver tumors. However, definite diagnosis cannot be obtained by histological evaluation alone in some cases, especially when the sample is a small biopsy; in such cases, immunohistochemical staining is very useful. Molecular pathological diagnosis involves various techniques—such as in situ hybridization, reverse transcription polymerase chain reaction (RT-PCR), and DNA sequencing; immunohistochemistry is the most frequently used technique due to its broad application, ease of performance and evaluation, and reasonable cost. The results of immunohistochemistry occasionally reflect specific genetic mutations. In this article, we provide an overview of the application of immunohistochemistry in the pathological diagnosis of liver tumors referring to the association with genetic alterations. Furthermore, we aimed to explain the practical points in the differential diagnosis of liver tumors by immunohistochemical staining.

## 2. Immunohistochemical Markers of Liver Tumors

### 2.1. Immunohistochemical Markers of Hepatocellular Carcinoma

#### 2.1.1. Markers of Hepatocellular Differentiation

##### Hepatocyte Paraffin 1

**Figure 1:**
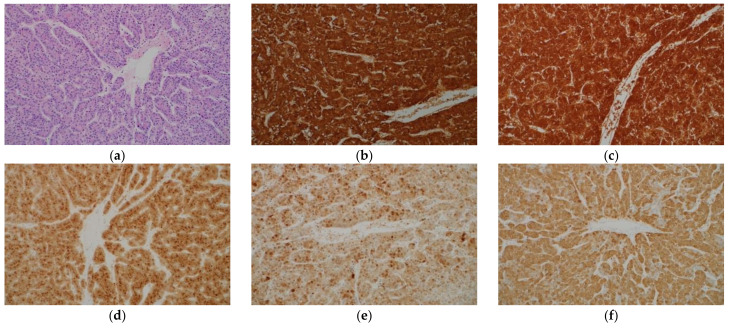
Histological appearance (**a**) and results of immunohistochemical staining (**b**–**f**) of hepatocellular carcinoma. (**a**) Tumor cells resembling hepatocytes proliferate, showing thick trabecular growth pattern. On immunohistochemistry, the tumor cells are positive for hepatocyte paraffin 1 (**b**), arginase-1 (**c**), glypican-3 (**d**), heat shock protein 70 (**e**), and glutamine synthetase (GS) (**f**). (Original magnification: ×200 for (**a**–**f**)).

Hepatocyte paraffin 1 (Hep Par 1) is a monoclonal antibody that was developed in 1993 using immunogens obtained from failed liver allografts (Table 1) [1]. It was later elucidated that the antigen for Hep Par 1 is the urea cycle enzyme carbamoyl phosphate synthetase 1 [2]. Hep Par 1 stains tumorous and non-tumorous hepatocytes and shows a diffuse cytoplasmic granular staining pattern (Figure 1a,b). The sensitivity of Hep Par 1 for the diagnosis of hepatocellular carcinoma (HCC) is more than 70%, and the specificity is also high [3,4,5,6,7]. However, there are several limitations in using this antibody for the diagnosis of HCC. First, it has a low sensitivity for diagnosing poorly differentiated HCC and scirrhous HCC [8,9]. Second, adenocarcinomas of various organs may show immunohistochemical positivity with Hep Par 1, although the frequency is low [4,6,10]. Finally, hepatoid carcinomas occurring in various organs often show immunohistochemical positivity with Hep Par 1 [11,12].

##### Arginase-1

Arginase-1 (Arg-1) is a binuclear manganese metalloenzyme mainly found in the liver that catalyzes the hydrolysis of arginine to ornithine and urea in the urea cycle [8,13]. Immunohistochemistry for Arg-1 stains both tumorous and non-tumorous hepatocytes and shows diffuse cytoplasmic staining pattern with variable nuclear reactivity (Figure 1c). Arg-1 is the most sensitive marker of HCC, with a high sensitivity even in poorly differentiated HCC and scirrhous HCC [8,9]. The specificity of Arg-1 immunohistochemistry for HCC is also high [13,14]. Positive staining is also observed in hepatoblastomas [15]. However, adenocarcinomas in various organs may show positivity for Arg-1, although the frequency is low [16], and hepatoid carcinomas occasionally exhibit positive staining for Arg-1 [12].

##### Polyclonal Carcinoembryonic Antigen

Carcinoembryonic antigen (CEA) is a glycoprotein found in the glycocalyx of fetal epithelial cells; a small amount of CEA is also observed in normal adult cells [8,17]. Polyclonal anti-CEA antibody (pCEA) cross-reacts with biliary glycoprotein and shows a characteristic canalicular staining pattern in normal liver tissue [18]. This canalicular staining pattern is maintained in many cases of HCC, with more than 70% cases showing positive staining [5,19]. Conversely, many adenocarcinomas, including cholangiocarcinoma, show diffuse cytoplasmic, membranous, and/or luminal staining patterns. In case of poorly differentiated HCC, the diagnostic sensitivity decreases, and the staining pattern may be cytoplasmic; therefore, pCEA staining has limited ability to differentially diagnose poorly differentiated HCC and adenocarcinoma [18,20]. Luminal or membranous staining in adenocarcinomas and canalicular staining in HCC may appear confusing to pathologists. In addition, canalicular staining patterns are occasionally difficult to recognize for unexperienced pathologists. Due to these shortcomings of pCEA, Hep Par 1, and Arg-1 are currently more frequently used than pCEA as immunohistochemical markers of HCC.

##### CD10, Villin, and Bile Salt Export Protein

CD10 shows a canalicular staining pattern in tumorous and non-tumorous hepatocytes, similar to that of pCEA staining; however, the sensitivity of immunohistochemical staining for CD10 for HCC diagnosis tends to be lower than that for pCEA [6,21,22]. The interpretation of staining is easier for CD10 than for pCEA because cytoplasmic staining is less frequently observed [18]. Immunohistochemistry for villin and bile salt export protein (BSEP) shows similar canalicular staining patterns in HCC [4,12,17].

#### 2.1.2. Markers of Malignant Hepatocytes

##### Glypican-3

Glypican-3 (GPC-3) is a heparan sulfate proteoglycan that is attached to the cell surface by a glycosyl-phosphatidylinositol anchor; it is highly expressed in embryonic tissues but has a low expression in normal adult tissues [23]. Positive staining for GPC-3 is observed in approximately 80–90% of HCC cases (Figure 1d), but negative in the normal liver, hepatocellular adenoma (HCA), focal nodular hyperplasia (FNH), and large regenerative nodule [24,25,26]. It has cytoplasmic, membranous, and canalicular staining patterns. It may be stained diffusely or focally, and the sensitivity of GPC-3 immunohistochemistry for the diagnosis of HCC is as low as approximately 50% with needle biopsy specimens [16,27]. The sensitivity for the diagnosis of moderately and poorly differentiated HCC exceeds 80%, whereas that for the diagnosis of well differentiated HCC is as low as approximately 60% [25]. This finding is important because Hep Par 1 immunostaining has a low sensitivity for diagnosing poorly differentiated HCC. It was reported that 100% of poorly differentiated HCCs could be detected by combining GPC-3 and Arg-1 [8]. The positive rate in scirrhous HCC is as high as approximately 80% [9]. However, the fact that cirrhotic nodules and active hepatitis C cases may stain positively for GPC-3 requires attention [24,28,29]. Furthermore, GPC-3 expression is frequently observed in hepatoblastoma and undifferentiated embryonal sarcoma [30,31], and dysplastic nodule (DN) may also show positive staining [25,32]. In addition to liver tumors, the expression of GPC-3 is frequently observed in squamous cell carcinoma of the lung, testicular non-seminomatous germ cell tumors, and liposarcoma [33].

##### Heat Shock Protein 70

Heat shock protein (HSP) 70 is an anti-apoptotic regulator that promotes cell survival and may be associated with tumorigenesis [34]. In a study of gene expression profiles in HCC, HSP70 was the most abundantly upregulated gene in early HCC [35]. Immunohistochemical examination showed that HSP70 expression was the highest in progressed HCC, followed by early HCC, and then, precancerous lesions, in that order [35]. The sensitivity and specificity of HSP70 immunostaining in the diagnosis of early and grade 1 HCC were 78% and 95%, respectively [36]. The staining pattern is usually patchy, and the nucleus and cytoplasm are stained; diffuse staining is observed in only one-third of the cases (Figure 1e) [18,36]. The bile duct epithelium is also stained, which can serve as an internal control. Special attention is required during pathologic evaluation since benign hepatocytes may also be stained. When non-tumorous tissue is also sampled, more intense staining in the tumorous tissue than in the non-tumorous tissue should be interpreted as positive staining. HCA cases show no immunostaining for HSP70 [37,38]. HSP70 is not useful in differentiating hepatocellular from non-hepatocellular neoplasms as it is frequently expressed in intrahepatic cholangiocarcinoma (ICC) and metastatic liver tumors [39].

##### Glutamine Synthetase

Glutamine synthetase (GS) catalyzes the synthesis of glutamine by promoting the condensation of glutamate and ammonia in the liver [34,40]. GS is the target of β-catenin and is upregulated when this pathway is activated. In the normal liver, the expression of GS is confined in two to three cell-thick hepatocytes around the central veins [41]. In a cirrhotic liver, this characteristic staining pattern is not maintained [18]. GS is a marker of HCC and immunostaining for GS is positive in 80% of low-grade HCC cases (Figure 1f) [37]. As described below, immunohistochemical staining for GS is also useful in the diagnosis of FNH and a certain type of HCA. GS immunostaining is not useful in differentiating hepatocellular from non-hepatocellular neoplasms as GS expression is observed in 76% of ICCs and 71% of metastatic liver tumors [39].

##### α-Fetoprotein

α-Fetoprotein (AFP) is an oncofetal protein produced by the liver and visceral endoderm of the yolk sac [17,20]. Although it is a marker of HCC, germ cell tumors, such as yolk sac tumor, also express this protein. Although serum AFP levels often increase in patients with HCC, the sensitivity of immunohistochemical staining for AFP for diagnosing HCC is as low as approximately 30%, and the staining pattern is patchy in many cases [4,6]. Therefore, immunohistochemical staining for AFP has limited utility in HCC diagnosis. Presently, immunohistochemical staining for AFP is less frequently performed for the diagnosis of HCC, because better diagnostic markers have been developed.

##### CD34

CD34 is useful for the diagnosis of liver tumors because it shows different staining patterns between tumorous and non-tumorous liver tissues. In a normal liver or a cirrhotic liver, immunohistochemistry for CD34 stains the endothelial cells of blood vessels in the portal tracts and the fibrous septa; however, the sinusoidal endothelial cells are not stained, except in the areas adjacent to the portal tracts and fibrous septa. Arterialization in HCC, HCA, and FNH induces the capillarization of sinusoids and sinusoidal endothelial cells stain positively for CD34. Diffuse staining of the sinusoidal endothelial cells is observed in almost all cases of HCC [26]. Many cases of HCA and FNH show incomplete staining patterns, with rare instances of diffuse staining patterns [26].

#### 2.1.3. Subtypes of Hepatocellular Carcinoma That Show Special Immunohistochemical Staining Patterns

##### Scirrhous Hepatocellular Carcinoma

Scirrhous HCC is a rare subtype of HCC characterized by prominent stromal fibrosis [9,42]. Other characteristics of scirrhous HCC include its subcapsular location, contiguous multinodular-type gross appearance, absence of capsule and necrosis, preserved portal tracts in the tumor, remarkable lymphocytic infiltration, clear cell change, and presence of hyaline bodies [42,43]. It was reported that the sensitivity of immunostaining with Hep Par 1 and pCEA for diagnosing scirrhous HCC was as low as 26% and 37%, respectively, while that of epithelial cell adhesion molecule (EpCAM), cytokeratin (CK) 19, and CK7 immunostaining was as high as 63%, 26%, and 53%, respectively [9]. Scirrhous HCC is prone to be misdiagnosed as ICC or metastatic adenocarcinoma due to the abundant stroma and the abovementioned immunohistochemical features. The sensitivity of GPC-3 and Arg-1 immunostaining for diagnosing scirrhous HCC have been reported to be as high as 79% and 85%, respectively, and it was 100% when these two markers were used in combination [9]. Therefore, immunohistochemical staining for GPC-3 and Arg-1 is useful for the diagnosis of scirrhous HCC.

##### Fibrolamellar Hepatocellular Carcinoma

This subtype of HCC is characterized by a lamellar pattern of fibrosis and presence of large tumor cells with abundant eosinophilic cytoplasm [44,45], and almost all cases express CK7 [46,47,48]. Fibrolamellar HCC usually demonstrates positive immunostaining with anti-Arg-1, Hep Par 1, and pCEA antibodies, but the positivity rate of GPC-3 immunostaining is rather low (17–64%) [25,44,45,47,48]. Although almost all fibrolamellar HCC cases show a distinctive granular, dot-like, or stippled pattern of cytoplasmic staining for CD68, the positivity rate in control HCCs was reported to be approximately 25% when the background liver was non-cirrhotic, and approximately 10% when the background liver was cirrhotic [49]. Therefore, the diagnosis of fibrolamellar HCC should be established cautiously when immunohistochemical staining for CK7 and CD68 is negative. The *DNAJB1-PRKACA* fusion gene, a result of ~400-kilobase deletions on chromosome 19, is characteristically found in fibrolamellar HCC; RT-PCR, fluorescence in situ hybridization, and RNA in situ hybridization are useful for its detection [50,51]. These molecular pathological techniques are useful in cases where fibrolamellar HCC is suspected but definite diagnosis cannot be made based on the histologic and immunohistochemical findings alone. However, it was recently reported that *DNAJB1-PRKACA* fusion is also observed in oncocytic pancreatic and biliary neoplasms and is not specific to fibrolamellar HCC [52].

### 2.2. Immunohistochemical Characteristics of Focal Nodular Hyperplasia

**Figure 2:**
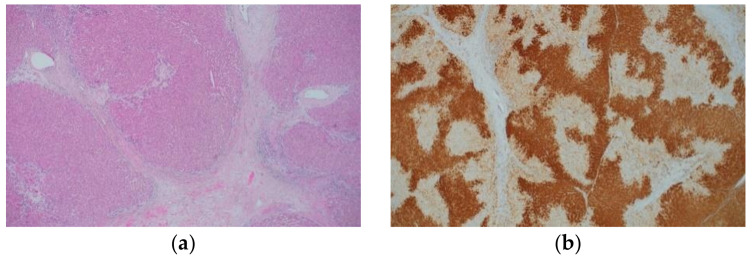
Histological appearance (**a**) and results of immunohistochemical staining (**b**) of focal nodular hyperplasia. (**a**) The lesion is characterized by thick fibrous septa with a central scar, thick-walled abnormal blood vessels, and ductular reaction. (**b**) Immunohistochemistry for GS shows a map-like staining pattern. (Original magnification: ×100 for (**a**,**b**)).

FNH is considered to be a hyperplastic lesion of the hepatocytes due to increased blood flow associated with vascular malformation [53]. FNH is characterized by nodular architecture, thick fibrous septa with or without a central scar, thick-walled abnormal blood vessels, and ductular reaction (Figure 2a and Table 2) [17,45,54]. FNH shows a characteristic map-like pattern in the immunohistochemical staining for GS (Figure 2b). Namely, large areas with positively stained hepatocytic cytoplasm anastomose, often surrounding the hepatic veins, and intermingle with small unstained areas that are close to the fibrous bands containing arteries and ductules [55]. This map-like pattern must be differentiated from the diffuse GS staining pattern, a characteristic of β-catenin activation; however, this differentiation may be difficult with small biopsy specimens. On the contrary, as aforementioned, the expression of GS is restricted to two to three cell-thick hepatocytes around the central veins in a normal liver. Positive staining for serum amyloid A (SAA) is observed in approximately 20% of FNH cases [56], and the map-like GS staining pattern is useful in differentiating FNH from inflammatory hepatocellular adenoma (IHCA) (described later). In FNH, activating mutations of *CTNNB1* (encoding β-catenin) are not observed despite the fact that the β-catenin pathway is activated [57,58]. The expansion of areas with GS-positive hepatocytes in FNH is attributed to this phenomenon.

### 2.3. Immunohistochemical Characteristics of Hepatocellular Adenoma 

HCA is a relatively rare benign tumor of hepatocytic origin. The risk factors for HCA include female sex, exposure to steroid sex hormones (oral contraceptives, anabolic steroids, and pregnancy), glycogenosis types 1 and 3, maturity onset diabetes of the young type 3 (MODY3), and familial polyposis coli [34,59,60]. Currently, advancements in molecular pathological studies have enabled the classification of HCA into several types (Table 2). Each type has unique morphological features, but examination using several immunohistochemical markers is indispensable for accurate classification.

#### 2.3.1. *Hepatocyte Nuclear Factor 1A*-Inactivated Hepatocellular Adenoma

**Figure 3:**
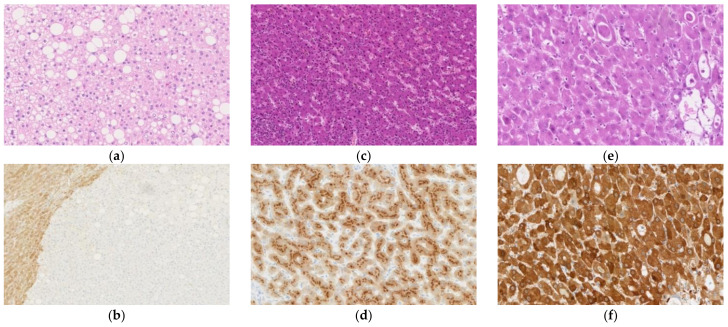
Histological appearance (**a**,**c**,**e**) and results of immunohistochemical staining (**b**,**d**,**f**) of hepatocellular adenoma (HCA). (**a**) *Hepatocyte nuclear factor (HNF) 1A*-inactivated HCA (H-HCA) shows prominent steatosis. (**b**) Immunohistochemical staining for liver fatty acid-binding protein is negative in the H-HCA lesion but positive in the surrounding liver tissue. (**c**) Inflammatory HCA (IHCA) is characterized by sinusoidal dilation and patchy inflammatory cell infiltration. (**d**) On immunohistochemistry, tumor cells of IHCA are positive for serum amyloid A. (**e**) β-catenin-activated HCA (b-HCA) may be associated with cellular and structural atypia, and pseudoglands may be observed. (**f**) b-HCAs with *CTNNB1* gene mutations that lead to strong β-catenin pathway activation show a strong/homogenous staining pattern for GS. (Original magnification: ×150 for (**a**,**d**), ×100 for (**b**,**c**), and ×200 for (**e**,**f**)).

This subtype is characterized by mutations in the *hepatocyte nuclear factor (HNF) 1A* gene. It mostly occurs in young women and presents with prominent steatosis (Figure 3a), although some cases lack this feature. Sinusoidal dilation and cellular atypia do not usually occur, and it rarely progresses to HCC [54,60,61]. Liver fatty acid-binding protein (LFABP) is downregulated, and immunohistochemistry for LFABP is negative because of mutation in the *HNF1A* gene, which encodes hepatocyte nuclear factor 1 (Figure 3b). In the normal liver and other types of HCA, the hepatocyte cytoplasm is stained in the immunohistochemistry for LFABP. Immunohistochemical staining for LFABP is not useful for differentiating HCA from HCC since the expression of LFABP may also be downregulated in HCC [62,63]; it is only useful for subclassification after a definite diagnosis of HCA. Immunohistochemical staining for SAA and C-reactive protein (CRP) as well as nuclear accumulation of β-catenin is usually negative, and GS immunostaining does not show a map-like or diffuse pattern [54].

#### 2.3.2. Inflammatory Hepatocellular Adenoma

This subtype is characterized by gene mutations that activate the interleukin (IL)-6 signaling pathway, and mutation of the *IL6ST* gene, which encodes the signaling co-receptor, gp130, occurs most frequently [17]. Mutations in *FRK*, *STAT3*, *GNAS*, and *JAK1* have also been reported. Histologically, IHCA is characterized by sinusoidal dilation, patchy inflammatory cell infiltration, and variable steatosis (Figure 3c) [54,60]. Differentiation between IHCA and FNH based on the histologic features alone is difficult as IHCA often possesses features common to FNH, such as fibrous septa and ductular reaction [56]. For this reason, IHCA was formerly called “telangiectatic FNH.” Almost all IHCA cases show strong and diffuse cytoplasmic immunostaining for SAA (Figure 3d) and CRP, both of which are proteins associated with inflammation [17,56,60]. However, 15% of FNH cases show diffuse staining for CRP [56]. IHCA is suggested if a map-like staining pattern is not observed on GS immunostaining and a diffuse staining pattern is observed for CRP immunostaining.

#### 2.3.3. β-Catenin-Activated Hepatocellular Adenoma

This subtype shows activation of the WNT signaling pathway, resulting from mutation or deletion of the *CTNNB1* gene, which encodes β-catenin [58]. This subtype may be associated with cellular and structural atypia, and pseudoglands may be observed (Figure 3e). The degree of β-catenin activation is associated with the mutation pattern of the *CTNNB1* gene: (1) S45, K335, and N387 mutations cause weak activation; (2) T41 mutations cause moderate activation; and (3) exon 3 deletions and amino acid substitutions within the β-TRCP binding site (D32-S37) cause a high degree of activation [64]. This is associated with the immunohistochemical staining pattern for GS. Tumors with mutations that lead to strong β-catenin activation show a strong/homogenous immunostaining pattern (Figure 3f), while tumors with mutations that lead to weak activation show a heterogeneous pattern, with the former being associated with malignancy [58]. Many cases of β-catenin-activated HCA (b-HCA) show nuclear staining in immunohistochemistry for β-catenin; however, the staining pattern is only focal and is not observed in some cases [58]. In the normal hepatocytes, β-catenin staining is observed at the sub-membranous location. Tumors with both IHCA and b-HCA features are called β-catenin-activated IHCA (b-IHCA), and the risk of malignant transformation of b-IHCA is similar to that of b-HCA with mutations in exon 3 of the *CTNNB1* gene [58]. Our group found that all HCA cases with nuclear accumulation of β-catenin showed preserved or increased expression of organic anion-transporting polypeptide (OATP) 1B3, while almost all HCA cases without nuclear accumulation of β-catenin showed decreased expression of OATP1B3 [65]. OATP1B3 is an organic anion transporter that contributes to the hepatocytic uptake of gadolinium-ethoxybenzyl-diethylenetriamine pentaacetic acid (Gd-EOB-DTPA), a hepatocyte-specific contrast agent used in magnetic resonance imaging (MRI) [66]. In accordance with the results of our previous study, the frequency of low signal intensity in the hepatobiliary phase of Gd-EOB-DTPA-MRI is lower in b-HCA than in other HCA subtypes [67].

#### 2.3.4. Other Types of Hepatocellular Adenoma

HCAs without characteristic pathological or genetic findings are diagnosed as unclassified HCA (UHCA). Henriet et al. [68] reported an upregulation of the arginine synthesis pathway, which is associated with the overexpression of argininosuccinate synthase 1 and arginosuccinate lyase in UHCA. Nault et al. [69] reported a subgroup of UHCA in which the sonic hedgehog signaling was activated by deletions that fused the promoter of *INHBE* with *GLI1*, and these tumors were associated with obesity and bleeding. The classification of HCA might change in the future through further molecular investigations.

### 2.4. Immunohistochemical Characteristics of Intrahepatic Cholangiocarcinoma

**Figure 4:**
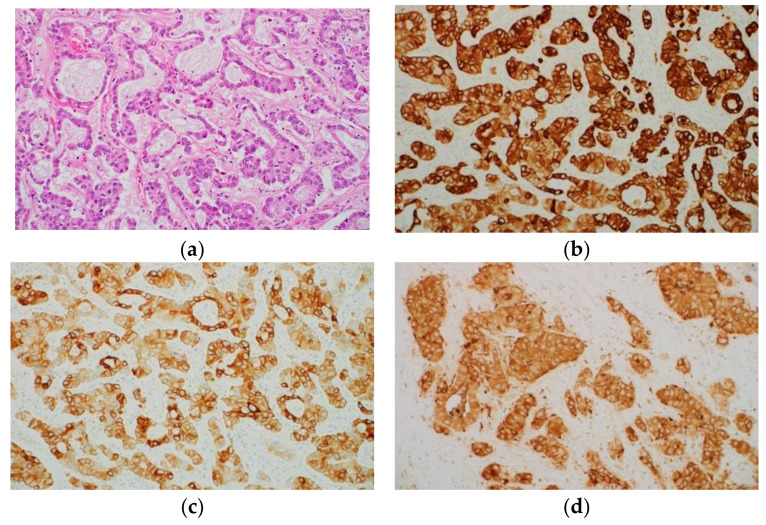
Histological appearance (**a**) and results of immunohistochemical staining (**b**–**d**) of intrahepatic cholangiocarcinoma (ICC). (**a**) Histologically, ICC is an adenocarcinoma. Immunohistochemically, tumor cells are positive for cytokeratin (CK) 7 (**b**), CK19 (**c**), and carcinoembryonic antigen (monoclonal antibody) (**d**). (Original magnification: ×200 for (**a**–**d**)).

Normal bile duct epithelial cells express CK7 and CK19, and almost all ICC cases also express these proteins (Figure 4a–c) [23,70]. Accordingly, CK7 and CK19 can be regarded as markers of biliary differentiation; however, morphologically pure HCC may show positive staining for CK7 and CK19 [71,72,73]. In other words, CK7 and CK19 are not specific markers of ICC. Immunostaining with Hep Par 1 is usually not observed in ICC. However, it was reported that the mucus-secreting cells in approximately 15% of ICC cases showed positive immunostaining with Hep Par 1 [74]. ICC tumor cells often show cytoplasmic and luminal positivity on immunohistochemical staining with monoclonal and polyclonal antibodies against CEA (Figure 4d). In addition, ICC and metastatic adenocarcinoma frequently show positive staining for EpCAM [4,5]. However, HCC cases rarely show positive staining for EpCAM [5,75,76].

ICC is classified into large and small duct types. The large duct type ICC is characterized by immunohistochemical expression of MUC5AC and MUC6, and *KRAS* mutations, while the small duct type ICC is characterized by immunohistochemical expression of CD56 and CRP, and *IDH1/2* mutations [58]. Recent studies on ICC suggest that there are molecular subclasses of proliferation and inflammation types in ICC that have different clinicopathological features and gene mutations [77], some of which may be candidates for targeted, personalized therapy. Furthermore, there appear to be associations between the inflammation subclass and the large duct type, and between the proliferation subclass and the small duct type. Cholangiolocellular carcinoma (CoCC) is a carcinoma characterized by the proliferation of small glands, resembling the bile ductules, and its status in the classification of liver tumors is not definite. Balitzer et al. [78] reported that results of immunohistochemical staining for CK19, SALL4, CD56, CD117, and epithelial membrane antigen (EMA) as well as the molecular features based on next-generation sequencing results were similar between ICC and CoCC; they argued that CoCC should be classified as a subtype of ICC. In fact, CoCC is classified as a subtype of ICC in the current World Health Organization (WHO) classification, although it was classified as a subtype of combined hepatocellular-cholangiocarcinoma in the previous WHO classification [58]. However, in our previous study, immunohistochemical staining for β6, β4, and α3 integrins was negative to weakly positive in most cases of CoCC and HCC and strongly positive in most cases of ICC, suggesting that the features of CoCC were similar to those of HCC but not ICC [79]. It is conceivable that further examination is necessary to determine the position of CoCC.

## 3. Practical Points in the Differential Diagnosis of Liver Tumors by Immunohistochemical Staining

### 3.1. Early Hepatocellular Carcinoma vs. Dysplastic Nodule

DN is considered a precursor lesion of HCC. DN typically occurs in the cirrhotic liver, is usually approximately 1 cm in size, and is macroscopically different from the surrounding cirrhotic nodules in terms of color and/or texture [18,80]. DN can be classified as either low-grade or high-grade. High-grade DN (HGDN) shows cytological and structural atypia, but the degree is insufficient for the diagnosis of HCC, and the reticulin framework is intact. However, differentiating between HGDNs and early HCCs by histomorphology is often difficult. Stromal invasion is an important finding to differentiate early HCC from DN and is associated with the loss of ductular reaction [81]. Therefore, immunohistochemical staining for CK7 or CK19 is useful in differentiating between the two lesions; the presence of ductular reaction in the tumor border suggests DN, while its absence suggests early HCC. Di Tommaso et al. [82] examined the utility of three immunohistochemical markers (HSP70, GPC-3, and GS) in the differential diagnosis of large regenerative nodule, DN, and HCC in biopsy specimens. Although 22% of HGDN cases showed immunohistochemical positivity for one marker, none of the HGDN cases showed positivity for two or three markers. By contrast, 60.8% of the HCC cases were positive for three markers, and 78.4% of the HCC cases were positive for two markers. When limited to very-well-differentiated HCC and well differentiated HCC, 57% of the cases showed positivity for three markers, while 72.9% of the cases showed positivity for two markers. The same group also examined the utility of the abovementioned three markers in surgical specimens [36]. When at least two of the markers were positive on immunostaining, the sensitivity and specificity for the detection of early and grade 1 HCC were 72% and 100%, respectively. The utility of this panel of markers in the diagnosis of early HCC was confirmed in a subsequent study [83]. Immunohistochemistry for detecting these three markers is especially useful in biopsy specimens because the evaluation of stromal invasion is difficult on biopsied specimens. Distributions of sinusoidal capillarization in DN are intermediate between those in cirrhotic nodules and HCC [84]; therefore, immunohistochemical staining for CD34 may also be helpful in the differential diagnosis of DN and HCC.

### 3.2. Well Differentiated Hepatocellular Carcinoma vs. Hepatocellular Adenoma

Histological differentiation between well differentiated HCC and HCA is often difficult, especially in biopsy specimens. HCA (especially b-HCA) may show cytological and structural atypia; however, the degree is generally milder than that seen in HCC, and the reticulin framework is intact on silver impregnation staining. By contrast, loss of the reticulin framework is observed in many cases of HCC. In addition, immunohistochemical examination is useful for differentiating between the two lesions. As aforementioned, approximately 80–90% of HCC cases demonstrate positive GPC-3 immunostaining, but HCA cases consistently do not stain for GPC-3 [24,25,26]. In addition, 78% of early and grade 1 HCC cases exhibit positive staining for HSP70 [36], but HCA cases are consistently negative for HSP70 [37,38]. However, the sensitivity of these markers for diagnosing HCC by immunohistochemistry is not high with biopsy specimens. GS is not useful in the differential diagnosis of HCC and HCA since it may be diffusely stained in both lesions.

### 3.3. Focal Nodular Hyperplasia vs. Hepatocellular Adenoma

The central scar of FNH may be unclear; in such cases, histopathological differentiation between FNH and HCA (especially IHCA) is difficult. Differentiation between FNH and HCA in biopsy specimens is especially difficult. The most useful immunohistochemical markers for the differential diagnosis are GS and SAA/CRP. As aforementioned, FNH shows a characteristic map-like staining pattern on GS immunostaining. On the other hand, in HCAs other than b-HCA and b-IHCA, GS staining is negative or positive around the veins or shows scattered patchy staining. b-HCA and b-IHCA show diffuse homogenous/heterogeneous staining for GS [58]. Immunohistochemical staining for SAA and CRP is usually negative in FNH but diffusely positive in IHCA [18,58]. However, a small number of FNH cases show diffuse staining for SAA and CRP [56]; thus, an overall evaluation, including the characterization of GS immunostaining pattern, is necessary.

### 3.4. Hepatocellular Carcinoma vs. Intrahepatic Cholangiocarcinoma

Histopathological differentiation between HCC and ICC is not difficult in well differentiated and moderately differentiated tumors. However, in poorly differentiated tumors, differentiation between HCC and ICC based on histopathology alone may be difficult. Immunohistochemical staining for hepatocyte markers, such as Hep Par 1 and Arg-1, and bile duct cell markers, such as CK7 and CK19, is useful for differentiating HCC from ICC. However, as aforementioned, ICC may show positivity for hepatocyte markers, while HCC may show positivity for bile duct cell markers, though at a low frequency. Therefore, it is necessary to examine several markers and perform an overall evaluation, including histologic evaluation.

### 3.5. Intrahepatic Cholangiocarcinoma vs. Metastatic Adenocarcinoma in the Liver

Differentiation between ICC and metastatic adenocarcinoma in the liver is often clinically important. ICC is typically CK7(+) and CK20(−), while colorectal adenocarcinoma is usually CK7(−) and CK20(+). Therefore, these immunohistochemical markers are useful for differentiating between ICC and hepatic metastasis of colorectal cancer. In addition, CDX2 is a highly sensitive and specific marker of intestinal adenocarcinoma and is useful for differentiating between the two lesions [85]. We showed that the incidence of c-*myc* amplification in differential polymerase chain reaction assay was significantly higher in hepatic metastasis of colorectal cancer than in ICC [86]. In the future, the utility of immunohistochemical staining for c-myc in the differential diagnosis should be examined. Adenocarcinoma of the breast is frequently positive for estrogen receptor, while adenocarcinoma of the lung is frequently positive for thyroid transcription factor 1 (TTF-1) and napsin A. ICC is essentially negative for these markers; thus, these markers are useful for differentiating between ICC and hepatic metastasis of breast cancer and lung cancer. However, differentiation between ICC and hepatic metastasis of adenocarcinoma of the stomach, extrahepatic bile duct, gallbladder, and pancreas is often difficult, even if immunohistochemical staining is performed. Lok et al. [87] examined the immunohistochemical features of 41 cases of ICC and 60 cases of pancreatic ductal adenocarcinoma and reported that the S100P(−)/pVHL(+)/MUC5AC(−)/CK17(−) pattern was indicative of ICC, while the S100P(+)/pVHL(−)/MUC5AC(+)/CK17(+) and S100P(+)/pVHL(−)/MUC5AC(−)/CK17(+) patterns were indicative of pancreatic ductal adenocarcinoma. In addition, CRP is considered a potent diagnostic marker for ICC. Yeh et al. [88] examined the utility of immunohistochemical staining for CRP in the differential diagnosis of ICC, other adenocarcinomas, and metastatic liver tumors and reported that the sensitivity and specificity for diagnosing ICC were 75.7% and 91.1%, respectively, in tissue microarray, while they were 93.3% and 88.2%, respectively, in whole tissue sections.

## 4. Conclusions

The development of immunohistochemistry, which is currently the most frequently used molecular pathology technique, has caused significant change in pathological diagnosis. Due to the collective efforts of researchers, extensive knowledge has been accumulated on useful immunohistochemical markers for the diagnosis of liver tumors. This has contributed not only to the improvement of the accuracy of routine pathological diagnosis, but also to the elucidation of the mechanisms underlying the occurrence and differentiation of liver tumors and prognosis prediction. Hence, further studies on immunohistochemical markers of liver tumors are warranted for further improvements in diagnosis and treatment. Using a molecular-driven selection of biomarkers, Calderaro et al. [89] recently identified endothelial-specific molecule 1 (ESM1) as a potential immunohistochemical marker of macrotrabecular-massive HCC (MTM-HCC). The discovery of new disease markers starting from a specific molecular signature could be a good strategy to develop new tools that can identify and discriminate different types of liver tumors.

## Figures and Tables

**Table 1:** Immunohistochemical markers of HCC.

Marker	Staining Pattern	Characteristics
Markers of Hepatocellular Differentiation		
Hepatocyte paraffin 1	Cytoplasmic	The sensitivity decreases in the diagnosis of poorly differentiated HCC and scirrhous HCC.
Arginase-1	Cytoplasmic with variable nuclear reactivity	This is the most sensitive marker of HCC and shows high sensitivity even in poorly differentiated HCC and scirrhous HCC.
pCEA	Canalicular	The sensitivity decreases in poorly differentiated HCC. The staining may be difficult to interpret.
CD10	Canalicular	The sensitivity tends to be lower compared to pCEA.
Markers of Malignant Hepatocytes		
Glypican-3	Cytoplasmic, membranous, and canalicular	The sensitivity is low in well differentiated HCC and high in moderately and poorly differentiated HCC.
Heat shock protein 70	Nuclear and cytoplasmic	The staining pattern is usually patchy. Benign hepatocytes may be stained.
Glutamine synthetase	Cytoplasmic	This cannot be used in the differential diagnosis between HCC and HCA, and between hepatocellular and non-hepatocellular neoplasms.
α-Fetoprotein	Cytoplasmic	The sensitivity is low.
CD34	Sinusoidal	The evaluation is subjective.

HCA, hepatocellular adenoma; HCC, hepatocellular carcinoma; pCEA, polyclonal carcinoembryonic antigen.

**Table 2:** Molecular findings, histological features, and immunohistochemical findings of FNH and HCA.

Item	FNH	H-HCA	IHCA	b-HCA
Molecular findings	Activation of the β-catenin pathway without mutations of *CTNNB1*	Biallelic mutations of *HNF1A*	Activation of the IL-6 signaling pathway due to mutations in *IL6ST*, *FRK*, *STAT3*, *GNAS*, and *JAK1*	Activation of the WNT signaling pathway due to mutation or deletion of *CTNNB1*
Histological features	Thick fibrous septa with or without a central scar, abnormal blood vessels, and ductular reaction	Prominent steatosis	Sinusoidal dilation, patchy inflammatory cell infiltration, and variable steatosis	Cellular and structural atypia and pseudoglands
Immunohistochemical findings				
LFABP	(+)	(−)	(+)	(+)
β-catenin	Membranous	Membranous	Membranous	Nuclear
GS	Map-like	Patchy with perivascular accentuation	Patchy with perivascular accentuation	Diffuse homogenous or heterogenous staining depending on the mutation type
SAA/CRP	Usually (−)	(−)	Strong and diffuse	(−)
OATP1B3	(+)	(−)	(−)	(+)

b-HCA, β-catenin-activated HCA; CRP, C-reactive protein; FNH, focal nodular hyperplasia; GS, glutamine synthetase; HCA, hepatocellular adenoma; H-HCA, *HNF1A*-inactivated HCA; IHCA, inflammatory HCA; IL, interleukin; LFABP, liver fatty acid-binding protein; OATP, organic anion-transporting polypeptide; SAA, serum amyloid A; (+), positive; (−), negative.
